# A Case of Intractable Vomiting: Was It the Celiac Artery Compression?

**DOI:** 10.7759/cureus.22483

**Published:** 2022-02-22

**Authors:** Nardine Abdelsayed, Kevin Parza, Mohamed Faris

**Affiliations:** 1 Department of Internal Medicine, Grand Strand Medical Center, Myrtle Beach, USA

**Keywords:** celiac artery compression syndrome, chronic cholecystitis, celiac axis syndrome, dunbar syndrome, celiac artery compression, medial arcuate ligament, intractable vomiting

## Abstract

Celiac artery compression syndrome is a rare and poorly understood condition. Compression of the celiac artery by the median arcuate ligament causes intractable nausea, vomiting, and postprandial pain. We present a case of a 55-year-old male with a 50-pound unintentional weight loss and intractable vomiting, whose symptoms persisted despite robotic median arcuate ligament release. He later returned to the emergency department where he was found to have a low gallbladder ejection fraction on imaging indicative of biliary dyskinesia, for which he underwent a cholecystectomy. Eventually, his symptoms improved, and he was able to return to his baseline body weight.

## Introduction

Celiac artery compression syndrome or median arcuate ligament syndrome (MALS) is a rare condition caused by the compression of the celiac artery by the median arcuate ligament (which is a fibrous band of the diaphragm). Symptoms include postprandial pain, vomiting, and weight loss [1]. It could easily be mistaken with chronic mesenteric ischemia by symptomatology. The treatment for MALS is surgical decompression of the median arcuate ligament [2], but unfortunately, results have been variable. Although this condition can cause long-term symptoms, it is a diagnosis of exclusion, and other diagnoses should be considered if symptoms do not resolve with treatment.

## Case presentation

We present a case of a 55-year-old Caucasian male with a medical history significant for hypertension, coronary artery disease (with percutaneous coronary intervention (PCI)), and subsequent five-vessel coronary artery bypass grafting (CABG)) who was admitted for intractable nausea, vomiting, and abdominal discomfort.

He endorsed an insidious onset of nonspecific symptoms, including early satiety resulting in significant unintentional weight loss of approximately 50 pounds over the last year. This progressed to a constellation of symptoms including nausea with non-bloody, non-bilious emesis to frequent postprandial vomiting. Over the last several months, he developed occasional postprandial periumbilical discomfort that lasted a few hours. He denied any alleviating factors. On review of systems, he denied fevers, chills, night sweats, dysphagia, gastroesophageal reflux, hematemesis, change in bowel habits, melena, or hematochezia.

His home medications were aspirin and clopidogrel. He had no relevant surgical history, except for a left hernia repair. His social history was pertinent for cigarette use with smoking cessation.

On admission, his vital signs were normal, except for a rapid heart rate of 101 beats per minute (normal range: 60-90 beats per minute). His laboratory findings are seen in Table 1 and included an elevated white blood cell count with normocytic anemia. He was also hypochloremic with an elevated anion gap and lactic acidosis. His glucose was elevated at 214 mg/dL with a hemoglobin A1c of 6.9, and his acetone was negative. The patient was started on intravenous fluids, antiemetics, and pain medications. The next morning, his white blood cell count trended down, his anion gap had closed, and his follow-up lactic acid was normal.

**Table 1:** Abnormal laboratory findings

	Laboratory value	Reference range
White blood cell count	15.7 k/mm^3^	3.7–10.1 k/mm^3^
Hemoglobin	13.8 g/dL	14–16.4 g/dL
Mean corpuscular volume	91 fL	81–95 fL
Chloride	90 mmol/L	96–107 mmol/L
Bicarbonate level	26 mmol/L	22–32 mmol/L
Anion gap	15 mEq/L	<11 mEq/L
Lactic acid	2.2 mmol/L	<2 mmol/L

**Figure 1:**
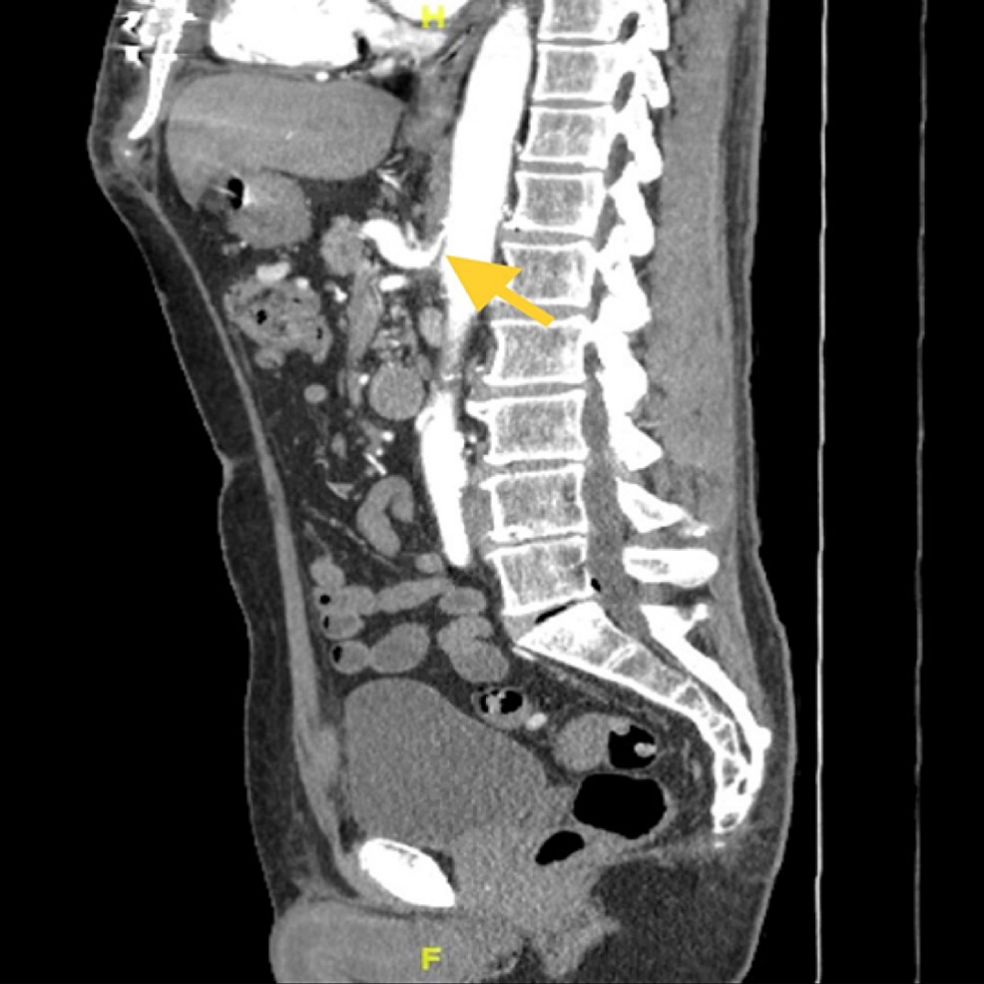
Sagittal view computed tomography angiography (CTA) of the abdomen and pelvis with a yellow arrow showing moderate median arcuate ligament compression of the celiac artery resulting in moderate ostial stenosis and post-stenotic dilatation

Gastroenterology consultation was placed for an esophagogastroduodenoscopy (EGD), which showed a small hiatal hernia and non-impacted Schatzi’s ring that which were not felt to be causing his current symptoms. Duodenal and gastric biopsies were obtained and were benign. He had a normal gastric emptying study. Colonoscopy showed diverticulosis. His computed tomography angiography (CTA) of the abdomen (Figure 1) showed compression of the celiac artery by the median arcuate ligament and uncomplicated diverticulosis. There were no hepatic or biliary abnormalities.

The patient underwent robotic-assisted arcuate ligament release as well as celiac plexus disruption with no complications. He initially improved and was able to tolerate oral intake. After diabetic education in the setting of new-onset diabetes mellitus, he was discharged home.

Unfortunately, his symptoms returned after two months. At that time, a hepatobiliary iminodiacetic acid (HIDA) scan showed a gallbladder ejection fraction of 30% (normal: >35%) and reproduced his pain. A laparoscopic cholecystectomy was then performed, which showed chronic cholecystitis. No calculi were noted, and the gallbladder was not cancerous. His nausea and vomiting continued to improve, and he eventually returned to his previous body weight at 10-month follow-up.

## Discussion

Celiac artery compression syndrome was first described many years ago, but surgical treatment was first discussed in 1963 by Harjole [3]. The fibrous median arcuate ligament can compress the celiac artery, limiting blood supply to the gut. Other anatomical abnormalities can also result in this syndrome, such as tumor compression. Patients may notice an improvement of symptoms during inspiration, due to a more vertical orientation of the celiac artery during inspiration, thus relieving the compression. Similarly, kyphotic posturing tends to improve symptoms, while lordosis tends to worsen them [1].

The key findings on the abdominal CTA include focal narrowing of the proximal celiac axis in a “hooked” appearance, as well as post-stenotic dilatation and occasionally collateral vascular vessels [1]. Color-coded duplex sonography appears to be more specific, showing higher blood flow velocity in the celiac artery during expiration than on inspiration [4]. This helps differentiate this disorder from fixed stenosis of the celiac artery.

Surgery is indicated in symptomatic patients or patients with greater than 50% stenosis of the celiac artery. Although a number of techniques have been cited in the literature, median arcuate ligament release has shown the most success. Jimenez et al. noted that 85% of patients had immediate postsurgical symptom relief and only 9.1% patients had symptom recurrence [5]. Other studies have been variable, however, showing as low as 46.1% of patients with symptom relief and as high as 38% with symptom recurrence [6]. Generally, intervention tends to improve the quality of life. However, psychiatric comorbidities are common among these patients and tend to predict worse quality of life outcomes after surgical intervention [7].

Unfortunately, some patients continue to experience symptoms despite surgery. Differentials should be examined, including pancreatitis, cholecystitis, gastritis, peptic ulcer disease, and mesenteric ischemia. Celiac plexus disruption or neurolysis, which is usually performed for pain associated with chronic pancreatitis and pancreatic malignancies, may provide symptomatic relief [8]. Given that psychiatric conditions worsen perceived quality of life as noted above, patients should be screened and treated for underlying psychiatric conditions.

## Conclusions

Although commonly seen as an incidental finding on imagining, true median arcuate ligament syndrome (MALS) is a rare condition with serious implications. Affected patients may develop intolerance to any oral intake, causing severe nutrient deficiencies and weight loss. The outcomes of laparoscopic treatment are varied but typically result in significant symptom improvement in patients who undergo the procedure. If symptoms fail to resolve following surgery, alternative diagnoses should be pursued. Unfortunately, a minority of patients continue to experience symptoms following treatment in the absence of other underlying disease processes.

## References

[1] Santos GM, Viarengo LM, Oliveira MD (2019). Celiac artery compression: Dunbar syndrome. J Vasc Bras.

[2] Khrucharoen U, Juo YY, Sanaiha Y, Chen Y, Jimenez JC, Dutson EP (2018). Robotic-assisted laparoscopic median arcuate ligament release: 7-year experience from a single tertiary care center. Surg Endosc.

[3] Harjola PT (1963). A rare obstruction of the coeliac artery. Report of a case. Ann Chir Gynaecol Fenn.

[4] Tembey RA, Bajaj AS, Wagle PK, Ansari AS (2015). Real-time ultrasound: key factor in identifying celiac artery compression syndrome. Indian J Radiol Imaging.

[5] Jimenez JC, Harlander-Locke M, Dutson EP (2012). Open and laparoscopic treatment of median arcuate ligament syndrome. J Vasc Surg.

[6] Sahm M, Otto R, Pross M, Scholbach T, Mantke R (2020). Laparoscopic therapy of the coeliac artery compression syndrome: a critical analysis of the current standard procedure. Ann R Coll Surg Engl.

[7] Skelly CL, Stiles-Shields C, Mak GZ (2018). The impact of psychiatric comorbidities on patient-reported surgical outcomes in adults treated for the median arcuate ligament syndrome. J Vasc Surg.

[8] Sachdev AH, Gress FG (2018). Celiac plexus block and neurolysis: a review. Gastrointest Endosc Clin N Am.

